# Young and Especially Senescent Endothelial Microvesicles Produce NADPH: The Fuel for Their Antioxidant Machinery

**DOI:** 10.1155/2018/3183794

**Published:** 2018-04-05

**Authors:** Guillermo Bodega, Matilde Alique, Lourdes Bohórquez, Miriam Morán, Luis Magro, Lilian Puebla, Sergio Ciordia, María C. Mena, Elvira Arza, Manuel R. Ramírez

**Affiliations:** ^1^Departamento de Biomedicina y Biotecnología, Universidad de Alcalá, Alcalá de Henares, 28805 Madrid, Spain; ^2^Departamento de Biología de Sistemas, Universidad de Alcalá, Alcalá de Henares, 28805 Madrid, Spain; ^3^Proteomics Facility, Centro Nacional de Biotecnología, CSIC, Campus de Cantoblanco, 28049 Madrid, Spain; ^4^Unidad Técnica de Microscopía, CNIC, C/ Melchor Fernández Almagro 3, 28029 Madrid, Spain

## Abstract

In a previous study, we demonstrated that endothelial microvesicles (eMVs) have a well-developed enzymatic team involved in reactive oxygen species detoxification. In the present paper, we demonstrate that eMVs can synthesize the reducing power (NAD(P)H) that nourishes this enzymatic team, especially those eMVs derived from senescent human umbilical vein endothelial cells. Moreover, we have demonstrated that the molecules that nourish the enzymatic machinery involved in NAD(P)H synthesis are blood plasma metabolites: lactate, pyruvate, glucose, glycerol, and branched-chain amino acids. Drastic biochemical changes are observed in senescent eMVs to optimize the synthesis of reducing power. Mitochondrial activity is diminished and the glycolytic pathway is modified to increase the activity of the pentose phosphate pathway. Different dehydrogenases involved in NADPH synthesis are also increased. Functional experiments have demonstrated that eMVs can synthesize NADPH. In addition, the existence of NADPH in eMVs was confirmed by mass spectrometry. Multiphoton confocal microscopy images corroborate the synthesis of reducing power in eMVs. In conclusion, our present and previous results demonstrate that eMVs can act as autonomous reactive oxygen species scavengers: they use blood metabolites to synthesize the NADPH that fuels their antioxidant machinery. Moreover, senescent eMVs have a stronger reactive oxygen species scavenging capacity than young eMVs.

## 1. Introduction

Extracellular vesicles are subcellular structures produced by many different cells and located in the extracellular compartment. There are diverse types of extracellular vesicles depending on their biogenesis, content, function, and/or biophysical properties [1, 2]. The most common classification includes three types: microvesicles (MVs) (also termed microparticles or ectosomes), exosomes, and apoptotic bodies [3]. MVs are released from the cell by the budding of the plasma membrane and represent not only a heterogeneous structural population, since their size usually ranges from 0.15 to 1 *μ*m or more, but also a heterogeneous functional group. They are present in all the body fluids: urine, saliva, bile, amniotic liquid, synovial, cerebrospinal, and seminal fluids as well as blood. Plasma MVs are of diverse cellular origin; they can arise from endothelial cells, erythrocytes, platelets, and leucocytes [4]. Blood MVs have been involved in the regular maintenance of endothelial cells [5] and other physiological functions such as coagulation, reticulocyte maturation, and angiogenesis [3] as well as mediators of endothelial dysfunction [6]. They have also been used as therapeutic tools in cardiovascular disease [7].

Reactive oxygen species (ROS) are produced as a consequence of cell metabolism, although the cells contain different mechanisms to eliminate them. In fact, the oxidative stress caused by an increase of ROS can be considered as an imbalance between ROS production and elimination. Although ROS have physiological roles in the regulation of vascular cell function [8], vascular formation, and development [9], this imbalance has been associated with aging and senescence [10–14] as well as other pathological conditions of the cardiovascular system [15–17], more specifically, with endothelial cell dysfunction [18–24].

NADPH is, obviously, closely related to NADH, another molecule with a reducing activity. Both may act as common mediators in different biological processes. Hence, it is common to find the term NAD(P)H, indicating that both molecules may be involved. Nevertheless, NAD^+^ is mainly involved in catabolic reactions and mitochondrial functions, whereas NADP^+^ is involved in cellular antioxidant systems and anabolic reactions [25]. In fact, NADPH is considered the sole source of reducing power of antioxidant systems [26]. This is the reason why NADPH is considered the “fuel” of the antioxidant machinery. We have recently demonstrated that endothelium-derived MVs (eMVs) have a well-developed and functional enzymatic team to eliminate ROS which, moreover, increases in senescence [27]. Apparently, if eMVs can eliminate ROS, they should (a) contain large amounts of NADPH, (b) obtain it from the plasma, or (c) be able to synthesize it. Thus, the aim of this work was to elucidate these questions. In addition, given the differences observed in our previous study, where senescent eMVs showed a higher capacity of ROS elimination than young eMVs, we have also analyzed the capacity of eMVs obtained from both young and senescent endothelial cells to produce NADPH.

## 2. Material and Methods

### 2.1. HUVEC Culture

Cryopreserved human umbilical vein endothelial cells (HUVECs) (ATCC Cat number PCS-100-010) were cultured in endothelial growth medium (Lonza) supplemented with 10% heat-inactivated foetal bovine serum (Sigma-Aldrich). Cultures were maintained at 37°C in a 5% CO_2_ atmosphere at 95% humidity. The HUVECs were serially passaged (the replicative senescence model). Cells passaged < 8 times (population doubling (PD) < 20; with PD calculated as [ln{number of cells harvested} − ln{number of cells seeded}/ln2]) were regarded as young endothelial cells, while those passaged 27–35 times (PD > 96) were regarded as senescent [28]. The proliferation rate of the latter cells is remarkably reduced, and more than 70% are positive for senescence-associated *β*-galactosidase. Prior to use, HUVEC extracts from cells passaged 4–8 (young pool) and from cells passaged 27–35 (senescent pool) were mixed (performed in quadruplicate).

### 2.2. Isolation and Characterization of Young and Senescent eMV

Young and senescent HUVEC-derived MVs were isolated from their culture medium. Briefly, samples were centrifuged using serial centrifugations (15 min at 3000 rpm, 30 min at 14,000 rpm), and pellets were frozen and stored at −20°C until use. Prior to use, MVs from cells passaged 4–8 (young pool) and from cells passaged 27–35 (senescent pool) were mixed (performed in quadruplicate).

MVs from a medium containing young and senescent HUVEC cells were characterized in terms of size using a Beckman Coulter Cytomics FC 500 flow cytometer running CXP software. MVs were considered to be those events gated with a size between 0.5–1.5 *μ*m; this gate was established from the side scatter versus forward scatter dot plot produced in a standardization experiment using the SPHERO™ Flow Cytometry Nano Fluorescent Size Standard Kit (Spherotech). The latter has size-calibrated fluorescent beads ranging from 0.1–1.9 *μ*m in diameter. Events below 0.2 *μ*m were excluded in order to adequately distinguish true events from the background; events > 1.9 *μ*m were excluded to prevent possible confusion with apoptotic bodies. The absolute number of MVs (events) per *μ*L was determined using Flow Count calibrator beads (Beckman Coulter) according to the manufacturer's recommendations and employing CXP software: (MVs counted × standard beads/L)/(standard beads counted). Data were recorded as the mean of three independent measurements of the same sample. The same number of senescent and young MVs was used in comparative analyses.

### 2.3. Western Blotting

The total protein content of extracts from young and senescent eMVs (performed using CytoBuster Protein Extraction Reagent lysis buffer (Millipore)), which contains a protease and a phosphatase inhibitor cocktail (Roche), was quantified using a BCA Protein Assay Kit (Pierce), employing BSA as the standard. Briefly, equal amounts of protein (25 *μ*g protein/lane) were diluted with a reducing sample buffer and separated by 4–20% Mini-PROTEAN® TGX™ Precast Protein Gels under reducing conditions. These proteins were then transferred onto nitrocellulose membranes (BioRad), blocked with TBS containing 0.1% Tween 20 and 5% dry nonfat milk for 1 h at room temperature, and incubated in the same buffer with different primary antibodies (anti-6PGL, Santa Cruz, sc-398833, dilution 1 : 500, 28 kDa; anti-GK, Santa Cruz, sc-393555, dilution 1/250, 61 kDa; anti-PSPH, Santa Cruz, sc-271421, dilution 1/250, 25 kDa). After washing with TBST, the membranes were incubated with Novex horseradish peroxidase-conjugated secondary antibodies followed by 2 additional washing steps with TBST. Bands were visualized with Luminata Crescendo Western HRP substrate (Millipore). Ponceau red (Sigma) staining was used as a loading control. Bands were quantified using Image J software (NIH) and normalized to Ponceau red.

### 2.4. Mass Spectrometry for NADPH Analysis

The presence of NADP^+^ and NADPH in eMVs was analyzed using a HPLC system Agilent 1100 coupled in-line to a TSQ Quantum triple quadrupole mass spectrometer (Thermo Scientific) equipped with an ESI source. Samples were prepared according to the “hot water/buffer extraction” protocol from Ortmayr [29]. Briefly, eMVs (50 × 10^6^) were added to 250 *μ*L of 5 mM ammonium acetate buffer at pH 8.0. The supernatant was collected after sample incubation for 3 min at 85°C, cooling on dry ice, and centrifugation for 10 min at 4000 ×*g*. Equipment settings were also obtained from Ortmayr [29] (see Table 1). Mass spectra were recorded in negative mode for NADP^+^ and NADPH. The column used was ACE Excel 3 C18 -PFP 150 mm × 3.0 mm + 3 *μ*m. Mass spectra of the column eluates were recorded in MS/MS mode using methanol and H_2_O, adding 5 mM ammonium acetate. Nitrogen was used as the ion source gas. The sheath gas pressure was set at 40 (arbitrary units), the auxiliary gas pressure was set at 2 (arbitrary units), and the spray voltage was set at 3000 V. The capillary temperature was set at 350°C. Argon was used as the collision gas for collision-induced dissociation at a pressure of 1.5 mTorr (Q2). Data were acquired using Xcalibur Control Software.

### 2.5. Proteomic Analysis

Proteomic analysis involved in-gel protein digestion followed by HPLC and mass spectrometry (MS). In order to obtain sufficiently large samples, the four HUVEC “young pool” extracts (see Section 2.2) were mixed, as were the four HUVEC “senescent pool” extracts, and the corresponding pools for the MVs. These four samples were dissolved in lysis buffer (8 M urea, 2 M thiourea, 5% CHAPS, 2 mM TCEP-HCl, and protease inhibitors). MVs were lysed by ultrasonication (10 strokes, low amplitude) on ice. The lysates were then centrifuged at 20,000 ×g for 10 min at 4°C, and the supernatant containing the solubilized proteins was used for LC-MS/MS experiments. Total protein concentration was determined using the Pierce 660 nm protein assay (Thermo). An aliquot of each sample was diluted with loading sample buffer and then loaded onto 1.2 cm wide wells of a conventional SDS-PAGE gel (1 mm thick, 4% stacking gel, and 12% resolving gel). The run was stopped as soon as the front entered 1 cm into the resolving gel, so that the whole proteome became concentrated at the stacking/resolving gel interface. The separated protein bands were visualized by Coomassie staining, excised, cut into cubes (cross section 1 mm^2^), deposited in 96-well plates, and processed automatically in a Proteineer DP (Bruker Daltonics). The digestion protocol used was based on Shevchenko et al. [30] with minor variations: gel plugs were washed firstly with 50 mM ammonium bicarbonate and secondly with acetonitrile prior to reduction with 10 mM dithiothreitol in 25 mM ammonium bicarbonate solution; alkylation was performed with 55 mM indoleacetic acid in 50 mM ammonium bicarbonate solution. The gel pieces were then rinsed, firstly with 50 mM ammonium bicarbonate and secondly with acetonitrile, and subsequently dried under a stream of nitrogen. Proteomics grade trypsin (Sigma-Aldrich) at a final concentration of 16 ng/*μ*L in 25% acetonitrile/50 mM ammonium bicarbonate solution was added and digestion allowed to proceed at 37°C for 4 h. The reaction was stopped by adding 50% acetonitrile/0.5% trifluoroacetic acid for peptide extraction. The tryptic-eluted peptides were dried by speed-vacuum centrifugation and then desalted on StageTip C18 Pipette tips (Thermo Scientific) until analysis by mass spectrometry.

A 1 *μ*g aliquot of each sample was subjected to 1D-nano LC ESI-MSMS analysis using an Eksigent Technologies nanoLC Ultra 1D plus nanoliquid chromatography system coupled to a high-speed Triple TOF 5600 mass spectrometer (SCIEX) with a Nanospray III source. The analytical column used was a silica-based reversed phase Acquity UPLC M-Class Peptide BEH C18 Column (75 *μ*m × 150 mm, 1.7 *μ*m particle size, and 130 Å pore size) (Waters). The trap column was a C18 Acclaim PepMap™ 100 (Thermo Scientific) (100 *μ*m × 2 cm, 5 *μ*m particle diameter, and 100 Å pore size), switched on-line with the analytical column. The loading pump delivered a solution of 0.1% formic acid in water at 2 *μ*L/min. The nanopump provided a flow-rate of 250 nL/min and was operated under gradient elution conditions. Peptides were separated using a 2–90% mobile phase B gradient (mobile phase A: 2% acetonitrile, 0.1% formic acid; mobile phase B: 100% acetonitrile, 0.1% formic acid) for 250 min. The injection volume was 5 *μ*L.

Data acquisition was performed with a Triple TOF 5600 System (SCIEX) (ionspray voltage floating 2300 V, curtain gas 35 psi, interface heater temperature 150°C, ion source gas 1 25 psi, and declustering potential 100 V). All data were acquired using information-dependent acquisition (IDA) mode with Analyst® TF 1.7 software (SCIEX). The following IDA parameters were chosen: a 0.25 s MS survey scan in the mass range 350–1250 Da, followed by 35 MS/MS scans of 100 ms in the mass range 100–1800 (total cycle time: 4 s). Switching criteria were set to ions greater than a mass to charge ratio (*m*/*z*) of 350 and smaller than 1250, with a charge state of 2–5 and an abundance threshold of more than 90 counts/s (cps). Former target ions were excluded for 15 s. The IDA rolling collision energy (CE) parameters script was used for automatically controlling the CE.

MS and MS/MS data obtained from individual samples were processed using Analyst TF 1.7 software. Raw data file conversion tools were used to generate mgf files which were then compared (using Mascot Server v.2.5.1 software; Matrix Science) to those in the UniProt *Homo sapiens* protein database. The latter contains 40,530 coding genes and their corresponding reversed entries. The search parameters were set as follows: carbamidomethyl (C) as the fixed modification and acetyl (protein N-term) and oxidation (M) as the variable modifications. Peptide mass tolerance was set to 25 ppm and 0.05 Da for fragment mass. Two missed cleavages were allowed. False discovery rates (≤1% at the spectral level) for peptide identification were calculated manually.

### 2.6. Functional Experiments

Two functional experiments were carried out in order to (1) test the ability of MVs to use blood metabolites to feed the NAD(P)H synthesis machinery, and (2) visually demonstrate that these metabolites induce NAD(P)H formation.

**Figure 1:**
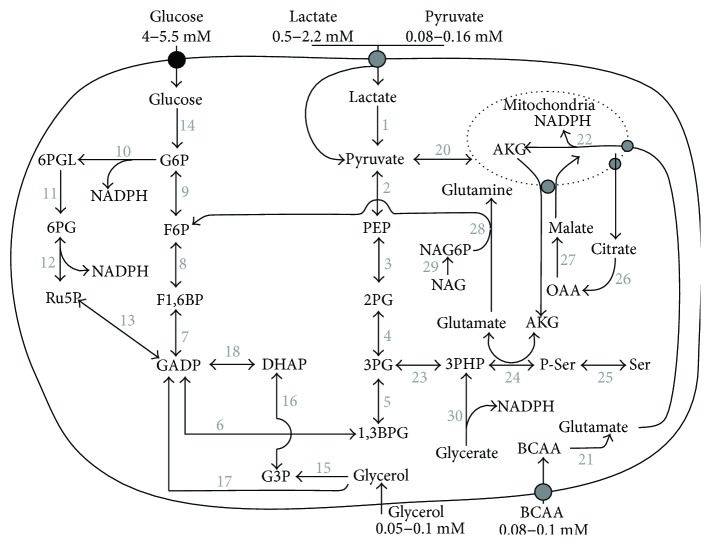
Schematic diagram of an eMV including the proposed metabolic pathways according to the proteomic results. The blood plasma metabolites that can feed these routes and their plasma concentrations under control conditions are also included. 1,3BPG: 1,3-bisphosphoglycerate; 2PG: 2-phosphoglycerate; 3PG: 3-phosphoglycerate; 3PHP: 3-phosphohydroxypyruvate; 6PG: 6-phosphogluconate; 6PGL: 6-phosphoglucono-1,5-lactone; AKG: alpha-ketoglutarate; BCAA: branched-chain amino acids; DHAP: dihydroxyacetone 3-phosphate; F1,6BP: fructose 1,6-biphosphate; F6P: fructose 6-phosphate; G3P: glycerol 3-phosphate; G6P: glucose 6-phosphate; GADP: glyceraldehyde 3-phosphate; NAG: N-acetylglucosamine; NAG6P: N-acetylglucosamine-6-phosphate; OAA: oxaloacetate; PEP: phosphoenolpyruvate; P-Ser: phosphoserine; Ru5P: ribulose 5-phosphate.

In the first experiment, five different blood metabolites were tested separately on young and senescent eMVs: lactate, pyruvate, glucose, branched-chain amino acids (BCAA; Val, Leu, Ileu), and glycerol. The concentration used was higher than the plasma value found in the bibliography (included in Figure 1): 7 mM, 0.5 mM, 15 mM, 0.3 mM, and 0.3 mM, respectively. In each tube, the test sample was formed by adding: 2 *μ*L of blood metabolite, 5 *μ*L of lysate including 10^6^ eMVs, and 5 *μ*L of 3 mM NADPH (Santa Cruz Biotechnology, reference number 202725A). No metabolites were included in the control samples. NADPH was used as the internal control; in fact, this experimental design allowed us to analyze the metabolite-induced changes in the NAD(P)^+^/NAD(P)H ratio. For example, for a certain metabolite, a lower ratio indicates that this metabolite increases the reduced form. After a 10 min incubation, 1 *μ*L of the sample was analyzed in a microvolume spectrophotometer (DeNovis DS-11 spectrophotometer), and the NAD(P)^+^/NAD(P)H ratio was determined by absorbance measurement at 260 nm/340 nm, respectively. The experiment was repeated three times using duplicated samples; twelve measures were performed in each case.

To visualize NAD(P)H synthesis, the eMVs (young and senescent) were incubated for 10 min with the blood metabolites previously described. Subsequently, 10 *μ*L of each sample including 40,000 eMVs was dropped into the center of a small water-repellent circle made on a slide with a PAP pen. The drop and the circle were wrapped up with a coverslip and observed in a Zeiss LSM 780 multiphoton confocal microscope with a Mai-Tai DS (690–1040 nm tunable) laser. The excitation wavelength was 735 nm, more than twice that of NAD(P)H (340 nm), and the laser intensity was set at 5.5%. The detection range was 396–502 nm.

### 2.7. Statistical Analysis

Data were analyzed using R software. The two-tailed Student *t*-test was used to analyze differences in Western blotting results. Data of our first functional experiment (ability of MVs to use blood metabolites to feed the NAD(P)H synthesis machinery) were analyzed as follows. Kruskal-Wallis test (with post hoc Dunn's test) was performed for the comparison of the effect of metabolites in young eMVs and senescent eMVs. Mann–Whitney–Wilcoxon rank-sum test was used to compare the effect of every molecule between young and senescent: glucose in young versus glucose in senescent and so on. Significance was set at *p* < 0.05.

## 3. Results

This section has been organized in three parts: (1) proteomic analysis, where we describe the biochemical pathways in eMVs which are directed at synthetizing NADPH; (2) the presence of NADP^+^ in eMVs; and (3) functional analysis, to demonstrate whether or not eMVs can synthesize NAD(P)H after activation of the pathways previously described. In the text that follows, the enzymes are named using the acronyms included in Table 2, the generic name (GN), and the metabolites with the acronyms included in Figure 1.

### 3.1. Proteomic Analysis

**Figure 2:**
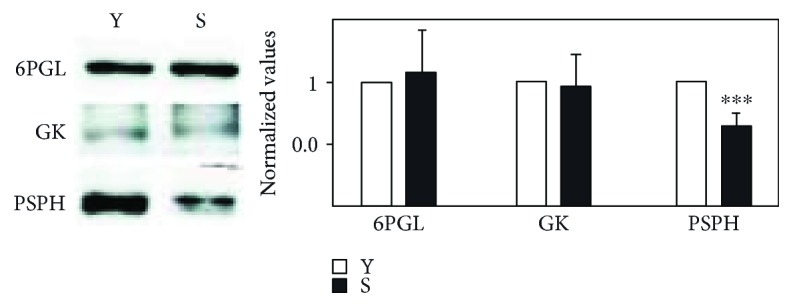
Western blot analysis of 6PGL, GK, and PSPH and the corresponding normalized analysis. A representative pool is included in the figure: young (Y) and senescent (S). THP-1 cells were used as a positive control (not shown). Bands were located at the expected molecular weight of 6PGL (28 kDa), GK (61 kDa), and PSPH (25 kDa). Protein data for the eMVs was normalized against the intensity of Ponceau red staining. Bars represent mean ± SD (*n* = 4 pools). ^∗∗∗^*p* < 0.001.

The pentose phosphate pathway (PPP) is the main biochemical route to synthesize NADPH. Hence, our first objective was to search for the presence of enzymes that participate in this pathway using mass spectrometry (MS) analysis of young and senescent eMVs. The majority of PPP enzymes were detected in this MS analysis, as shown in Table 2, except for 6PGL, GK, and PSPH, which were detected by Western blot (WB) analysis (Figure 2).

We next carried out a more detailed analysis of the enzymes detected in our MS results in order to connect the PPP with other biochemical routes. Table 2 includes the enzymes of the PPP and those of related metabolic routes proposed according to our proteomic analysis. To clarify these routes, a diagram of all the metabolic pathways proposed is included in Figure 1.

The organization of the different enzymes found in our proteomic analysis led us not only to establish the hypothetical biochemical routes that may be operative in the eMVs but also to point out the metabolites that feed these biochemical routes (Figure 1). All in all, it seems that both young and especially senescent eMVs have a very well-developed enzymatic organization designed to optimize NADPH production, and this is mainly due to the bidirectionality of most of these enzymatic activities. Besides the glycolytic pathways, which use glucose, lactate, and pyruvate, it is very important to point out the possibility that BCAA and glycerol can also be used to direct metabolites to the PPP. Mitochondria are also involved in this strategic metabolic organization: they can act as an NADPH biosynthetic organelle (note the high content of GLUD1 (number 22)) and supply AKG to the serine and 3PHP pathway. Enzymes involved in serine and glycerate metabolism are also included in these biochemical pathways using 3PHP and 3PG as metabolic intermediates.

One of the most interesting results was the higher content of most of these enzymes in senescent eMVs when compared with young eMVs; in fact, their enzymatic machinery seems to be designed to direct metabolites to the PPP or to obtain NADPH from new routes not included in young eMVs. The bold numbers in Table 2 indicate an increase > 25% when young and senescent eMVs were compared. A route specially used in senescent eMVs is the serine and PHP pathway, as suggested by the high content of the enzymes involved (numbers from 23 to 27) in senescent eMVs. The use of glutamine to form F6P using GFPT2 (number 28) is also especially elevated in senescent eMVs. Moreover, at the bottom of Table 2, we have included five enzymes that synthesize NADPH but are not included in Figure 1. Note that IDH1 and IDH2 are decreased in senescent eMVs, whereas the last three enzymes are increased.

### 3.2. Functional Analysis

We carried out two types of functional analysis; one to test the capacity of the proposed blood metabolites to feed the enzymatic machinery involved in reducing power synthesis and the other one to visualize this reducing power in eMVs. Unfortunately, NADH and NADPH are closely related molecules whose absorption and emission spectra are almost equal and, thereby, it has been impossible for us to separate NADH from NADPH in these functional analyses.

**Figure 3:**
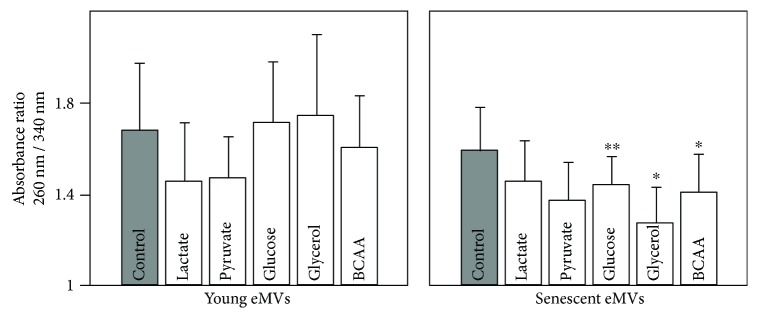
Effect of incubating eMVs with different blood plasma metabolites on the ratio of absorbance at 260 nm and 340 nm, absorption peaks of NAD(P) and NAD(P)H, respectively. No blood metabolites were added to control eMVs. Error bars represent SD; *n* = 3. To clarify the plot, only statistical significance of the Mann–Whitney–Wilcoxon rank-sum test is included. ^∗^*p* < 0.05, ^∗∗^*p* < 0.01. In the Kruskall-Wallis test for young eMVs, significant differences were observed in control versus lactate and pyruvate (*p* < 0.05) and between glucose versus lactate (*p* < 0.01). Significant differences were also observed for senescent eMVs in control versus glycerol and pyruvate (*p* < 0.05) and between glycerol versus glucose and lactate (*p* < 0.01).

In order to study the effect of blood metabolites on NAD(P)H synthesis, eMVs were incubated 30 min with the proposed metabolites (lactate, pyruvate, glucose, glycerol, and BCCA). These metabolites were incubated separately at a concentration that doubled the maximal value indicated in Figure 1. The absorbance ratio 260 nm/340 nm is a measure of the relative proportion of NAD(P)^+^/NAD(P)H. In young eMVs, lactate and pyruvate diminished significantly this ratio, which indicates that both metabolites induced the formation of NAD(P)H. However, in senescent eMVs, all the metabolites tested induced a decrease in this ratio, glycerol and pyruvate being statistically significant. Statistically significant differences were also observed for glucose, glycerol, and BCAA when the effect of metabolites was compared in young and senescent eMVs (Figure 3).

**Figure 4:**
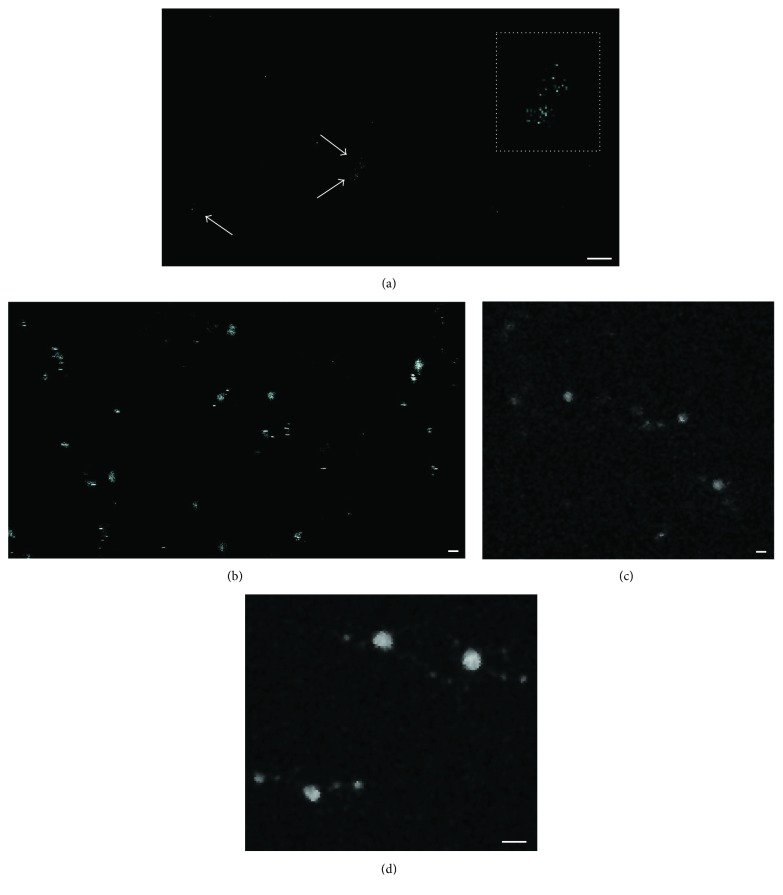
eMVs observed with a multiphoton confocal microscope. (a) Young eMVs under control conditions; arrows indicate eMVs. The square delimited by a dotted line is a magnification of the two eMVs indicated by the two arrows. (b) Senescent eMVs after glycerol incubation. (c) Young eMVs after lactate incubation. (d) Senescent eMVs after pyruvate incubation. The different size of the eMVs in the image is due to the fact that the eMVs were floating in the buffer in different positions in the *z*-axis. eMVs were obtained after mixing four pools. Scale bar, 1 *μ*m.

An experiment using multiphoton confocal microscopy was performed to visualize the production of NAD(P)H by eMVs after incubation with the previously used metabolites. In order to detect only the reduced forms, eMVs were excited at 340 nm (670 nm in the multiphoton confocal microscope), and emission was detected at 460 nm. Only after incubation with the proposed blood metabolites could eMVs be clearly visualized, demonstrating that eMVs have the capacity to synthesize reducing power (NAD(P)H) (Figure 4), although not all vesicles were able to produce fluorescence. Only lactate and pyruvate induced fluorescence in young eMVs, and senescent eMVs seemed to be more efficient in producing fluorescence.

### 3.3. NADP^+^ and NADPH in eMVs

**Figure 5:**
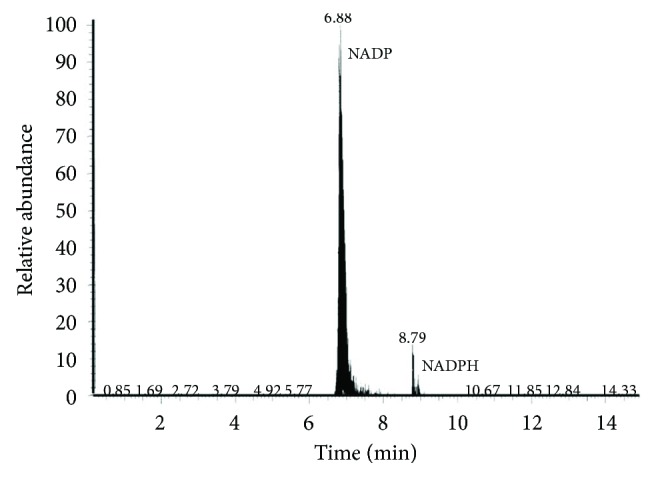
MS analysis of NADP^+^ (6.88 elution time) and NADPH (8.79 elution time) content in senescent eMVs. eMVs were obtained after mixing four pools. NADP^+^ MW: 742 D, NADPH MW: 744 D.

An MS analysis was carried out to accurately detect the presence of NADP^+^ and NADPH in eMVs. As can be seen in Figure 5, the presence of both cofactors was demonstrated in eMVs, although the content of NADP^+^ was greater than that of the reduced form. Differences in the NADP^+^/NADPH ratio were not observed either following incubation with the blood metabolites or between young and senescent eMVs.

## 4. Discussion

**Figure 6:**
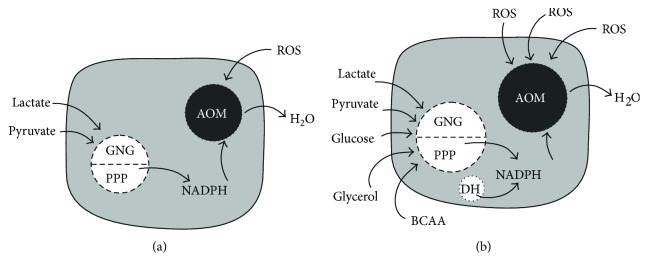
A young (a) and a senescent (b) eMV. AOM: antioxidant machinery; PPP: pentose phosphate pathway; GNG: gluconeogenesis; ROS: reactive oxygen species; DH: dehydrogenases; BCAA: branched-chain amino acids. Note that the senescent eMV has larger enzymatic machineries, uses more metabolites to feed them, and also has a higher capacity for ROS scavenging.

In a recent work, we demonstrated the existence of a functional machinery for ROS detoxification in HUVEC eMVs [27]; now, our present study demonstrates that these HUVEC eMVs, especially those derived from senescent cells, can synthesize NAD(P)H, the fuel that feeds the antioxidant machinery, used as precursors of different blood plasma metabolites. The combination of both results indicates that eMVs are subcellular structures with an autonomous capacity for ROS scavenging, as had been suggested by Soleti et al. [31]; eMV protection against oxidative stress regulating eNOS/Akt signaling has also been recently demonstrated [32].  Figure 6 shows a schematic drawing that represents our past and present results on the role of eMVs as ROS scavengers.

The presence of some antioxidant enzymes in plasma MVs [33–35], in endothelial-derived MVs [36], and in HUVEC-derived MVs [31] had been already demonstrated. However, in our previous work [27], we demonstrated that HUVECs had a complete antioxidant machinery and that their MVs included a specific group of functional enzymes mainly involved in O_2_^−^ and H_2_O_2_ detoxification. Moreover, to date, the possibility of NADPH synthesis in MVs has not been considered in the literature. As far as we know, this is the first time that the capacity of NADPH synthesis is ascribed to eMVs, although the existence of some PPP enzymes has been demonstrated in exosomes [37], and some enzymes included in Table 2 have also been found in different proteomic studies: numbers 1, 5, 6, 7, and 18 and IDH2 [36]; numbers 5, 6, and 7 [33]; numbers 6 and 7 and ALDH [35]; and numbers 10, 20, and 27 and ALDH [34]. The synthesis of NADPH in eMVs seems logical; it makes no sense to contain an antioxidant machinery without the capacity of synthesizing the molecule that feeds it.

NADPH is considered the essential reductant for antioxidant systems. Unfortunately, its absorption and emission spectra are very similar to that of NADH. The appearance of FLIM [38, 39] and MS [29] methods, however, has made it possible to study these metabolites separately. FLIM permits the monitoring of MVs, but the use of MS is ideal for quantitative comparative studies of oxidized (NADP^+^) and reduced (NADPH) forms. eMVs have an important handicap: it is difficult and very expensive to obtain large amounts of eMVs using HUVEC. This handicap has made our attempts to determine the NADP^+^/NADPH ratio using the standard commercial kits for NADPH analysis impossible. This problem can be resolved using FLIM, but this technique is very expensive and not usually available in most laboratories. While it is true that MS does not require big amounts of sample, even so, it is complicated to obtain enough eMVs for MS, and this problem gets worse in endothelial senescent cells because it is not easy to reach PD > 96. Note that the volume of 50 × 10^6^ eMVs can be, more or less, 0.025 mm^3^ of sample. Moreover, NADPH is not very stable [40]; heat specially induces its oxidation to NADP^+^, and MS samples have to be intensely heated up. This might explain not only the low signal of NADPH but also the lack of differences observed in our MS analysis after incubation with blood metabolites or between young and senescent eMVs. In addition, NADPH has a lower—one order of magnitude—response factor than NADP^+^. The filtering and dilution of the samples, as well as the existence of carbohydrates in the molecule (the ribose rings), that makes MS analysis difficult, must also be considered.

In this work, we propose a model of organization of the metabolic routes in eMVs in accordance with our proteomic analysis (MS and WB) (see Figure 1). Although both young and senescent eMVs can synthesize reducing power, the latter have a stronger synthetic machinery. In fact, it seems that senescent eMVs redesign their metabolic machinery to optimize NADPH synthesis. This new metabolic reorganization has some interesting hallmarks. Mitochondrial activity is deeply affected in order to diminish its activity: (1) ADP/ATP translocases diminish or disappear: translocase 1 from 362 (young eMVs) to 0 (senescent eMVs) (protein score values), translocase 2 from 580 to 102, and translocase 3 from 549 to 0; (2) mitochondrial aminotransferases also diminish or disappear: ornithine aminotransferase from 55 to 0, aspartate aminotransferase from 520 to 251, and serine hydroxymethyltransferase from 339 to 122; (3) pyruvate dehydrogenase which contributes to transforming pyruvate into acetyl-CoA, a metabolite used in the tricarboxylic acid (TCA) cycle, is diminished in senescent eMVs (see enzyme number 20 in Table 2); and (4) the TCA enzymes are unbalanced; for example, succinic dehydrogenase is not present in senescent eMVs, whereas isocitrate dehydrogenase and aconitase are diminished. The incorporation of pyruvate to the glycolytic pathway (note that PK (number 2) is augmented) and the sequestering of AKG in the serine and glutamine pathways in order to synthesize 3PG and F6P are in accordance with this depletion of mitochondrial metabolic activity.

It is well known that the main enzymes involved in NADPH synthesis are PPP dehydrogenases [41], isocitrate dehydrogenases, aldehyde dehydrogenases, malic enzyme, and NAD kinase [42]. PPP dehydrogenases (enzyme numbers 10 and 12) and alcohol (enzyme number 17) and aldehyde dehydrogenases are increased in senescent eMVs, as well as NAD(P)H dehydrogenase and flavin reductase (see the last three enzymes of Table 2). Moreover, two other dehydrogenases, GLUD1 (number 22), involved in mitochondrial NADPH synthesis, and LDH (number 1) (NAD-dependent), are strongly increased. Isocitrate dehydrogenase, however, is diminished (see above paragraph), and malic enzyme and NAD kinase have not been detected. A serine-related folate-dependent NADPH production has been demonstrated in proliferating [43] and in cancer [44] cells; however, the enzymes of this route have not been detected in our study.

Under high oxidative conditions, the PPP consumes more glucose to compensate for the depleted GSH [45]; in fact, a high oxidative stress drives G6P to the PPP, generating NADPH for antioxidant defenses [46], and a mechanistic link between increased G6PD (number 10) activity, elevated NADPH, and improved antioxidant protection has been suggested [47]. An activation of the PPP has also been demonstrated in cancer cells, probably to produce more NADPH to combat oxidative stress [48]; indeed, the existence of PPP enzymes has been demonstrated in exosomes of ovarian cancer cells in proteomic analysis [37]. Human skin cells also activate the PPP in response to oxidative stress [49], and senescent fibroblasts show increased glycolysis and PPP to reduce ROS production, upregulation of pathways involved in redox homeostasis [50], and alterations in nicotinamide metabolism [51]. Moreover, another different metabolic possibility has been suggested for ROS scavenging; the enzyme biliverdin reductase has been involved in a ROS-scavenging mechanism in a subpopulation of epididymal fluid MVs, protecting spermatozoa against ROS released from dying cells [52].

Evidence that MVs are a heterogeneous population is continuously increasing [53]. This heterogeneity can be considered at least from two different points of view: structural (size) and functional (biochemical). In our functional analysis using multiphoton confocal microscopy, not all MVs emitted fluorescence after their stimulation. In fact, we think that only a part of the eMVs could act as ROS scavengers. We have just seen that to accomplish this function, it is necessary to reorganize the metabolic routes of the eMVs, and it is logical to assume that this drastic reorganization may not be useful for other functions supported by MVs; for example, for those MVs capable of ROS synthesis and involved in signaling processes [54–56].

eMVs, or at least a subpopulation of them, produce reducing power, but the enzymatic machinery involved in this process also needs to be fed. In this study, we have demonstrated that lactate, pyruvate, glucose, glycerol, and BCAA fuel this reducing power synthetic machinery. All these metabolites are present in blood plasma and, except glycerol that diffuses freely, need membrane transporters to cross the plasma membrane and get into the MVs. Lactic acid can also diffuse freely; however, in the blood, it is dissociated and present as lactate (ionized) that cannot pass through the plasma membrane. The use of these metabolites is different in young (only use lactate and pyruvate) and senescent eMVs (use all). Obviously, the higher reducing power synthesis capacity of senescent cells is in accordance with their wider metabolite availability. On the other hand, the use of some of these metabolites could represent another metabolic advantage; for example, the use of lactate may serve to regulate lactate concentration and to prevent lactic acidosis and redox regulation in plasma. We have not detected monocarboxylate transporters (lactate and pyruvate transporters), neutral amino acid transporters, or glucose transporters in our proteomic study; however, the fluorescence emission after incubation of the eMVs with these metabolites implies the existence of a transport system. A low expression of these transporters could explain this result, but the fast (a few minutes) emission of fluorescence in eMVs after incubation with these metabolites suggests the contrary. The possibility that the membrane proteins, due to their hydrophobicity, are harder to detect in MS, indeed requiring thiourea to solubilize them, should be considered.

The contribution of ROS to aging has been widely documented, although the beneficial effects produced by ROS have led to a new interpretation of this traditional view [57–59]. An increase of ROS production has also been related with senescence, an experimental model widely used in aging studies [20, 24, 60] and also demonstrated in our previous work in senescent endothelial cells [27]. In accordance with the stronger capacity of ROS elimination demonstrated by senescent eMVs in our previous work, the present study demonstrates a strong increase of the machinery involved in NADPH synthesis in senescent eMVs. However, the stronger capacity of senescent endothelial cells and their MVs to eliminate ROS and the higher ROS production observed in senescence seems paradoxical. Their increased capacity for ROS scavenging might be an adaptive mechanism to the higher oxidative stress of senescent cells, and it could be considered a strategy used by senescent cells to promote cell survival. Unfortunately, this increased ROS scavenging is unable to compensate the higher levels of oxidative stress associated with senescence. On the other hand, eMVs can not only eliminate ROS but can also be effective in protecting against oxidative stress, suppressing NADPH oxidase and ROS production [32]. Finally, it should be considered that these results are obtained using the *in vitro* replicative senescent model in HUVEC; undoubtedly, this is a starting point that needs to be confirmed in *in vivo* aging models.

## 5. Conclusions

Our present and previous results demonstrate that eMVs can act as autonomous ROS scavengers: they use blood metabolites to synthesize NADPH that fuels their antioxidant machinery; moreover, senescent eMVs have a stronger ROS-scavenging capacity. Whether alterations in the ability of eMVs to regulate the oxidant/antioxidant balance can act as etiopathogenic mechanisms in diseases associated to a misbalance in oxidative stress is left out of this study but probably merits to be considered in order to use these MVs as diagnostic tools and/or therapeutic targets.

## Figures and Tables

**Table 1:** Compound optimization table in MS/MS mode.

	NADP^+^	NADPH
Molecular formula in MS	C_21_H_27_N_7_O_17_P_3_	C_21_H_29_N_7_O_17_P_3_
Parent mass	742 *m*/*z*	744 *m*/*z*
Product ion (*m*/*z*)/collision energy	619.96 *m*/*z*/17 v	426.16 *m*/*z*/35 v
	407.89 *m*/*z*/34 v	407.96 *m*/*z*/36 v
	272.82 *m*/*z*/38 v	396.96 *m*/*z*/32 v

**Table 2:** Proteomic analysis of young and senescent eMVs. PS: protein score; PSM: peptide-spectrum match; NP: number of peptides (MS/MS scores are sums for the validated peptides assigned to each protein); **C**: coverage. PS, PSM, and NP are usually considered quantitative variables in the proteomic analysis. Bold numbers in the “senescent columns” indicate a 25% increase in senescent eMVs; italic numbers in “young columns” indicate a 25% reduction in senescent eMVs. The 5 proteins at the bottom of the table are the enzymes that synthesize NADPH. UniProt: UniProt code; GN: generic name.

Enzymes		UniProt	GN	Young eMVs				Senescent eMVs			
				PS	PSM	NP	**C**%	PS	PSM	NP	**C**%
**1**	l-Lactate dehydrogenase B chain	**P07195**	**LDHB**	644	17	10	32	**837**	**18**	**12**	33.8
	l-Lactate dehydrogenase A chain	**P00338**	**LDHA**	458	14	7	25.6	**1023**	**26**	**15**	**53.6**
	l-Lactate dehydrogenase A-like 6A	**Q6ZMR3**	**LDHAL6A**	104	2	1	6.3	105	2	1	6.3
	l-Lactate dehydrogenase A-like 6B	**Q9BYZ2**	**LDHAL6B**					**38**	**1**	**1**	**4.5**
**2**	Pyruvate kinase	**P14618**	**PKM**	1425	40	24	56.9	**1675**	**44**	24	60.8
**3**	Alpha-enolase	**P06733**	**ENO1**	810	14	10	49.8	**1376**	**31**	**18**	**60.4**
	Gamma-enolase	**P09104**	**ENO2**	288	6	3	13.8	**542**	**10**	**7**	**38.5**
	Beta-enolase	**P13929**	**ENO3**	319	6	3	13.6	**425**	**9**	**5**	**18.7**
**4**	Phosphoglycerate mutase 1	**P18669**	**PGAM1**	197	6	3	33.5	**352**	**11**	**6**	**44.5**
**5**	Phosphoglycerate kinase 1	**P00558**	**PGK1**	524	12	7	46.5	519	12	8	45.6
**6**	Glyceraldehyde-3-phosphate dehydrogenase	**P04406**	**GAPDH**	1645	50	23	75.5	**1939**	**65**	**27**	72.2
**7**	Fructose-bisphosphate aldolase A	**P04075**	**ALDOA**	565	12	9	44.2	**1202**	**29**	**19**	**59.9**
	Fructose-bisphosphate aldolase C	**P09972**	**ALDOC**	310	5	4	25.5	**474**	**9**	**7**	25.3
**8**	Fructose-2,6-bisphosphatase	**Q9NQ88**	**TIGAR**					**46**	**1**	**1**	**6.7**
**9**	Glucose-6-phosphate isomerase	**P06744**	**GPI**					**272**	**6**	**5**	**20.6**
**10**	Glucose-6-phosphate 1-dehydrogenase	**P11413**	**G6PD**					**45**	**1**	**1**	**11.8**
**11**	6-Phosphogluconolactonase	**O95336**	**6PGL**	Western blot							
**12**	6-Phosphogluconate dehydrogenase, decarboxylating	**P52209**	**PGD**	162	3	3	22.2	160	3	3	24.2
**13**	Transketolase	**P29401**	**TKT**	**238**	7	4	**14.4**	185	6	4	19.3
**14**	Hexokinase-1	**P19367**	**HK1**	265	5	5	16.4	262	7	5	9.6
**15**	Glycerol kinase	**P32189**	**GK**	Western blot							
**16**	Glycerol-3-phosphate dehydrogenase, mitochondrial	**P43304**	**GPD2**	52	1	1	39				
**17**	Alcohol dehydrogenase [NADP(+)]	**P14550**	**AKR1A1**					**44**	**1**	**1**	**7.1**
**18**	Triosephosphate isomerase	**P60174**	**TPI1**	434	8	7	52.4	**523**	**12**	8	54.9
**19**	Glutamine-fructose-6-phosphate aminotransferase	**O94808**	**GFPT2**	43	1	1	4.3	**84**	**4**	**2**	4.4
	Glutamine-fructose-6-phosphate aminotransferase	**Q06210**	**GFPT1**					**241**	**4**	**4**	8.9
**20**	Pyruvate dehydrogenase E1 component subunit alpha	**P08559**	**PDHA1**	**5**	**3**	**13.8**	**80**	2	1	5.4	
	Pyruvate dehydrogenase E1 component subunit beta	**P11177**	**PDHB**	**114**	**2**	**2**	**17.3**	38	1	1	9.7
**21**	Branched-chain amino acid aminotransferase, mitochondria	**O15382**	**BCAT2**					**94**	**1**	**1**	**6.4**
**22**	Glutamate dehydrogenase 1, mitochondrial	**P00367**	**GLUD1**	329.0	11.0	5.0	24.0	**637.0**	**17.0**	**11.0**	**35.5**
**23**	d-3-Phosphoglycerate dehydrogenase	**O43175**	**PHGDH**	80	2	1	5.1	**391**	**8**	**6**	**23.3**
**24**	Phosphoserine aminotransferase	**Q9Y617**	**PSAT1**					**47**	**1**	**1**	**5.4**
**25**	Phosphoserine phosphatase	**PSPH**	**P78330**	Western blot							
**26**	ATP-citrate synthase	**P53396**	**ACLY**	286	6	5	16.7	**358**	**8**	**6**	**13.4**
**27**	Malate dehydrogenase, cytoplasmic	**P40925**	**MDH1**	99	3	1	10.8	**176**	**5**	**3**	**17.7**
**28**	Glutamine-fructose-6-phosphate aminotransferase	**O94808**	**GFPT2**	43	1	1	4.3	**84**	**4**	**2**	**4.4**
**29**	N-Acetyl-d-glucosamine kinase	**Q9UJ70**	**NAGK**					**193**	**3**	**3**	**22.1**
**30**	Glyoxylate reductase/hydroxypyruvate reductase	**Q9UBQ7**	**GRHPR**					**38**	**1**	**1**	**3**

Other NAD(P)H-related enzymes											
	Isocitrate dehydrogenase (NADP) cytoplasmic	**O75874**	**IDH1**	**41**	**1**	**1**	**2.7**		
	Isocitrate dehydrogenase (NADP), mitochondrial	**P48735**	**IDH2**	**254.0**	**6.0**	**5.0**	**38.7**	133.0	3.0	3.0	30.8
	Flavin reductase (NADPH) Alpha-aminoadipic semialdehyde	**P30043**	**BLVRB**					**37**	**1**	**1**	**12.1**
	Alpha-aminoadipic semialdehyde dehydrogenase	**P49419**	**ALDH7A1**	111	2	2	11.9	**207**	**5**	**4**	**16.5**
	NAD(P)H dehydrogenase (quinone)	**P15559**	**NQO1**					**39**	**1**	**1**	**6.6**
